# MiR-653-5p drives osteoarthritis pathogenesis by modulating chondrocyte senescence

**DOI:** 10.1186/s13075-024-03334-5

**Published:** 2024-05-29

**Authors:** Yucheng Lin, Lu Zhang, Mingliang Ji, Sinuo Shen, Yuzhi Chen, Shichao Wu, Xiaotao Wu, Nancy Q. Liu, Jun Lu

**Affiliations:** 1 Department of Orthopaedic Surgery, Zhongda Hospital, School of Medicine, Southeast University, Nanjing, 210009 Jiangsu People’s Republic of China; 2 Department of Anesthesiology, Women’s Hospital of Nanjing Medical University, Nanjing Maternity and Child Health Care Hospital, Nanjing, 210004, Jiangsu People’s Republic of China; 3 Department of Biochemistry and Molecular Biology, Wayne State University of Medicine, Detroit, MI 48201, USA; 4 Department of Orthopaedic Surgery, Keck School of Medicine of USC, University of Southern California (USC), Los Angeles, CA 90033 USA

**Keywords:** Osteoarthritis, Chondrocyte senescence, Articular cartilage, miR-653-5p, Pathogenesis, IL-6/JAK/STAT3 signaling pathway

## Abstract

**Background:**

Due to the unclear pathogenesis of osteoarthritis (OA), effective treatment for this ailment is presently unavailable. Accumulating evidence points to chondrocyte senescence as a key driver in OA development. This study aims to identify OA-specific microRNAs (miRNAs) targeting chondrocyte senescence to alleviate OA progression.

**Methods:**

We screened and identified miRNAs differentially expressed in OA and normal cartilage, then confirmed the impact of miR-653-5p on chondrocyte functions and senescence phenotypes through in vitro experiments with overexpression/silencing. We identified interleukin 6 (IL-6) as the target gene of miR-653-5p and confirmed the regulatory influence of miR-653-5p on the IL-6/JAK/STAT3 signaling pathway through gain/loss-of-function studies. Finally, we assessed the therapeutic efficacy of miR-653-5p on OA using a mouse model with destabilization of the medial meniscus.

**Results:**

MiR-653-5p was significantly downregulated in cartilage tissues and chondrocytes from OA patients. Overexpression of miR-653-5p promoted chondrocyte matrix synthesis and proliferation while inhibiting chondrocyte senescence. Furthermore, bioinformatics target prediction and the luciferase reporter assays identified IL-6 as a target of miR-653-5p. Western blot assays demonstrated that miR-653-5p overexpression inhibited the protein expression of IL-6, the phosphorylation of JAK1 and STAT3, and the expression of chondrocyte senescence phenotypes by regulating the IL-6/JAK/STAT3 signaling pathway. More importantly, the cartilage destruction was significantly alleviated and chondrocyte senescence phenotypes were remarkably decreased in the OA mouse model treated by agomiR-653-5p compared to the control mice.

**Conclusions:**

MiR-653-5p showed a significant decrease in cartilage tissues of individuals with OA, leading to an upregulation of chondrocyte senescence phenotypes in the articular cartilage. AgomiR-653-5p emerges as a potential treatment approach for OA. These findings provide further insight into the role of miR-653-5p in chondrocyte senescence and the pathogenesis of OA.

**Supplementary Information:**

The online version contains supplementary material available at 10.1186/s13075-024-03334-5.

## Background

Osteoarthritis (OA), the most common joint disease worldwide, imposes substantial mental and physical burdens on elderly individuals [1]. With aging, its prevalence keeps increasing. OA is characterized by the progressive deterioration of articular cartilage, resulting in diminished joint mobility and functionality [2, 3]. Currently, no drugs have been approved for OA modification and available interventions are limited to pain relief, leading to inevitable joint replacement surgery for patients with advanced OA [4]. This phenomenon is due to a poor understanding of the pathogenesis of OA. Joint trauma, obesity, aging and inflammation may all play a role in the progression of OA, which result in structural deterioration and failure of synovial joints [5, 6].

To date, although the interdependent relationship with many other risk factors may exist, aging has always been considered an essential risk element for OA, which is characterized by cellular senescence [7]. Chondrocytes constitute the primary cellular component within articular cartilage, and they play a pivotal role in sustaining the dynamic balance between extracellular matrix anabolism and catabolism [8]. However, senescent chondrocytes are less responsive to anabolic cytokines and are more sensitive to catabolic cytokines and secrete various inflammatory cytokines known as the senescence-associated secretory phenotype (SASP) [9]. The release of SASP and the senescence of chondrocytes may exacerbate the catabolic inflammatory environment, thereby indirectly worsening the already compromised reparative capabilities of articular cartilage [10]. Intriguingly, senescent chondrocytes impair cartilage homeostasis in isolated human articular cartilage chondrocytes from donors aged ranging from 1 to 87 years [11], which provides evidence to suggest that chondrocyte senescence will be a common molecular mechanism underlying both age-related and post-traumatic OA [6, 12].

Therefore, rational regulation of chondrocyte senescence may prevent or reverse OA processes. MicroRNAs (miRNAs) are small evolutionarily conserved non-coding RNAs (18–25 nt in length), which maintain cellular function by fine-tuning multiple genes expressions, and their dysregulation is associated with various human diseases [13]. Mounting evidence has strongly elucidated the physiological and pathogenetic role of miRNAs in the regulation of joint homeostasis and the development of OA [14]. Thus, the establishment of miRNA expression profiles and screening of miRNAs that target the senescence phenotype of chondrocytes are essential for investigating the underlying mechanisms of OA.

In this study, we extensively profiled miRNAs using NGS and found a significant downregulation of miR-653-5p in OA cartilage tissues compared to normal controls. Subsequently, we systematically validated the role of miR-653-5p in a series of experiments performed in the culture of human chondrocytes and C28/I2 cells. Furthermore, bioinformatics target prediction and the luciferase reporter assays identified interleukin 6 (IL-6) as a target of miR-653-5p. Moreover, in vitro and in vivo assays showed that upregulation of miR-653-5p significantly inhibited the senescence phenotype of chondrocytes and reduced cartilage destruction by targeting the IL-6/JAK/STAT3 signaling pathway. Our findings provide further insight into the role of miRNA in chondrocyte senescence and the pathogenesis of OA. MiR-653-5p may be a novel therapeutic target for developing new OA therapeutic strategies.

## Methods

### Patient samples

A total of 71 human OA cartilages and 33 normal cartilages were obtained from individuals undergoing knee arthroplasty and trauma patients without the history of OA or rheumatic arthritis, respectively. OA was diagnosed according to the American College of Rheumatology criteria [15]. All cartilage samples were obtained from the medial side of the knee joint. The specimens were further processed for histological examination and were categorized according to the modified Mankin scoring system [16]. Of them, three cartilage specimens of each group (pathological vs. control) were randomly selected for miRNA solexa sequencing. The Ethics Committee of our institution approved this study protocol, and written informed consent was obtained from each participant.

### MicroRNA-sequencing analysis

The Illumina HiSeq 2000 platform (San Diego, CA, USA) was employed to purify three OA patients and three normal controls for miRNA-sequencing analysis, following the manufacturer’s directions. The OA and control samples showed homogeneity in the selection of demographic factors, including gender, age, and body mass index. MiRNA sequencing was conducted utilizing miRBase 21.0. For quality control and library preparation for Illumina sequencing, a minimum quantity of 500 ng of total RNA was necessary. Following the purification of tiny RNA molecules (less than 30 nucleotides) using PAGE purification, a pair of solexa adaptors were ligated to their 5′ and 3′ ends. The amplification of small RNA molecules was conducted employing adaptor primers for a total of 17 cycles. Subsequently, fragments with an estimated length of 90 bp, which included both the small RNA and adaptors, were extracted from an agarose gel. The DNA that had undergone purification was utilized for the purpose of cluster creation and subsequent sequencing analysis. The image files created by the sequencer were then subjected to processing in order to provide data of high digital quality. Following the masking of adaptor sequences and elimination of contaminated reads, the clean reads underwent computational analysis.

#### Cell culture and transfection

Chondrocytes were extracted as described before [17]. Primary human chondrocytes were acquired from human OA cartilage (damaged medial femoral condyle) and normal controls. The articular cartilage of the medial femoral condyle in mouse knee with OA was dissected employing a surgical microscope, with the objective of precisely isolating the cartilage while avoiding the underlying subchondral bone. Dissected articular cartilage was subjected to enzymatic digestion to acquire the primary chondrocytes. In brief, PBS was utilized to wash the articular cartilage that underwent dissection, and then it was subjected to a 15-min incubation at 37 °C in trypsin-ethylenediaminetetraacetic acid (EDTA). This was followed by utilizing 2 mg/mL collagenase at 37 °C for 2-h digestion in Dulbecco’s modified Eagle’s medium treated with 10% fetal bovine serum (FBS), 100 U/mL penicillin, and 100 mg/mL streptomycin in a 5% CO_2_ atmosphere. Throughout the culturing time, cells were maintained at 37 °C in an environment of 5% CO_2_ and 95% air, with medium changes occurring every 2–3 days. The identical medium as OA chondrocytes was employed to preserve the C28/I2 cells.

Human chondrocytes and C28/I2 cells underwent transfection with miR-653-5p mimics labeled or unlabeled with Cy3 utilizing the Silencer® siRNA Labeling Kit (AM1636), miR-653-5p inhibitor, and their negative controls (Thermo Scientific Dharmacon ®) at 50 nM utilizing Lipofectamine RNAiMAX Transfection Reagent (Invitrogen, Life Technologies, CA, USA). Then, the cells were utilized for the subsequent investigations at 48 h (normal chondrocytes) or 72 h (OA chondrocytes) following the transfection. The establishment of OA cells of C28/I2 cell line used the method of IL-1 (1.5 ng/L, Sigma Aldrich, Mo, USA) stimulation. The cells were employed for subsequent experiments after stimulating the cells with IL-1 for one day.

### RNA isolation, cDNA synthesis, and quantitative real-time PCR analysis (qRT-PCR)

MiRNeasy Mini Kit (Qiagen, Valencia, CA, USA) was utilized to isolate the total RNA from cartilage tissues and cultivated cells following the manufacturer’s directions. Nanodrop (Thermo Scientific, Waltham, MA, USA) and Bioanalyzer (Agilent Inc., Santa Clara, CA, USA) were employed to detect the quantity and quality of RNA. TaqMan microRNA Reverse Transcription Kit (Applied Biosystems, Foster City, CA, USA) in a final volume of 15 µl was employed to generate cDNA (16 °C for 30 min, 42 °C for 30 min, 85 °C for 5 min, and hold at 4 °C). The experimental procedure included conducting all reactions in triplicate utilizing a 7500 real-time system (Applied Biosystems, CA, USA). The reactions were performed under specific conditions, which included a first denaturation step for a duration of 10 min at 95 °C, 40 cycles of denaturation at 95 °C for 15 s, and subsequent annealing/extension for a period of one minute at 60 °C. The comparative Ct (ΔΔCt) technique (2^−ΔΔCt^ with logarithm transformation) was utilized to conduct data analysis. The specific primers are as follows: miR-653-5p: 5′-GTGTTGAAACAATCTCTACTG-3′ and 5′-TCCACGACACGCACTGGATACGAC-3′, U6 snRNA: 5′-GTGCTCGCTTCGGCAGCACAT-3′ and 5′-TACCTTGCGAAGTGCTTAAAC-3′.

### 5-Ethynyl-2′-deoxyuridine (EdU) assay

EdU assay with a Kit of EdU Staining Proliferation and Alexa Fluor 555 (Beyotime, Shanghai, China) was employed to detect Cell proliferation depending on the direction of the manufacturer. Concisely, chondrocytes were introduced into 24-well plates at a density of 2 × 10^5^ per well. The plates were then incubated at 37 °C in 5% CO_2_. Following this, a concentration of 50 µM of EdU was administered to each well for 2 h. Following that, Hoechst 33,258 (Beyotime, Shanghai, China) was utilized to stain the cells. The fluorescence microscope (Olympus, Japan) was used to evaluate the ratio between EdU-positive cells and total Hoechst 33,258-positive cells.

### Senescence-associated β-galactosidase (SA-β-Gal) assay

The SA-β-Gal staining was conducted employing the cell senescence β-galactosidase staining kit (Beyotime Biotechnology, China), based on the guidelines provided by the manufacturer. In a concise manner, the cells underwent a washing process utilizing PBS and were subsequently fixed with a solution comprising 2% paraformaldehyde and 0.2% glutaraldehyde for 5 min. Then, the cells underwent a washing procedure and were exposed to a staining solution containing SA-β-Gal for 16 h at 37 °C. Following the incubation, the cells underwent a process of washing, and a Nikon Eclipse Ni-U microscope was utilized to image the cells.

### Fluorescence in situ hybridization (FISH)

A locked nucleic acid probe with complementarity to miR-653-5p was labeled with 5′ and 3′-digoxigenin, and Exiqon (Woburn, MA, USA) was utilized for synthesis. The chondrocytes from OA patients and normal controls were employed for FISH detection. After seeding the cells on glass slides to complete the sample preparation, FISH Tag RNA Multicolor Kit (F32956, Alexa Fluor™ dye combination, Invitrogen, USA) was utilized for the detection based on the manufacturer’s direction.

A PNA probe (Panagene, F1006) was utilized to conduct Telomere FISH analysis. Chondrocytes were seeded onto glass slides in six-well culture plates and subjected to 2 h incubation at 37°. The adhered cells then received KCl buffer treatment to induce swelling, fixed in a solution of methanol and acetic acid (3:1), rehydrated in PBS, and subsequently, 4% formaldehyde was utilized for fixation. Dehydration was achieved by a sequential application of ethanol concentrations. The slides were then exposed to a hybridization mixture comprising 10 mM NaHPO4 (pH 7.4), 10 mM NaCl, 70% formamide, and 20 mM Tris’s buffer (pH 7.5). To denature the chromosomal DNA, the slides were positioned for 5 min on an 80 °C heating block. Subsequently, the PNA probe was applied to the slides and subjected to 2 h incubation at room temperature. After a comprehensive cleaning process, the slides were prepared for examination by being mounted with Vectashield mounting media that included 4,6-diamidino-2-phenylindole (DAPI) (Vector Labs). Subsequently, a confocal microscope (Carl Zeiss, Oberkochen, Germany) was employed to analyze the slides.

Mitochondrial transmembrane potential was measured by FISH using the JC-1 probe. JC-1 monomers were green fluorescence, and JC-1 mitochondrial aggregates were red fluorescence. This FISH test was conducted utilizing a mitochondrial membrane potential assay kit (Beyotime Biotechnology, China). For JC-1 staining, 1 × 10^6^ cells were subjected to 10 min incubation with 10 mg/ml JC-1 37 °C, and a confocal microscope (Carl Zeiss, Oberkochen, Germany) was utilized to analyze the cells for red and green fluorescence.

### 3′-Untranslated region (UTR) cloning and luciferase assay

To generate the wild-type (WT) IL-6 3′UTR-Luc reporter plasmid (IL-6 3′UTR), we amplified a fragment of the IL-6 gene’s 3′UTR, which contained the predicted miR-653-5p binding location, via PCR. Subsequently, the fragment that underwent amplification was cloned downstream of the firefly luciferase gene in the psi-CHECK^TM^-2 vector (Promega, Madison, WI) utilizing XhoI and NotI enzymes (Thermo Fisher Scientific). To create constructs with mutations in the putative miR-653-5p binding location of the WT IL-6 3′UTR, the QuikChange Lightning Site-Directed Mutagenesis Kit (Agilent Technologies, CA, USA) was employed to conduct site-directed mutagenesis. Following the PCR, a 20 µL portion of the reaction mixture underwent digestion with DpnI at 37 °C for one hour. Subsequently, a 10 µL fraction was subjected to transformation into DH5 alpha Escherichia coli in order to generate the mutant construct plasmids. The confirmation of the authenticity of all constructions was achieved via the process of sequencing (Cosmogenetech, Seoul, Korea). In the luciferase examination, human primary chondrocytes and C28/I2 cells were evenly seeded and distributed at a concentration of 3000 cells per well in a 96-well plate. The cells were subjected to co-transfection with either the WT or mutant-type IL-6 3′UTR-Luc reporter plasmid, along with either the miR-control or miR-653-5p, employing Lipofectamine PLUSTM reagent (Invitrogen). Cell lysates were obtained two days post-transfection, and luciferase activity was quantified employing the Dual-Glo Luciferase Assay kit (Promega, WI, USA), depending on the guidelines of the manufacturer. The firefly luciferase activity was employed to standardize the luciferase activity.

### Western blotting

The Western blot analysis was conducted in accordance with established protocols. In this study, proteins were subjected to separation on a 10% SDS-PAGE gel. Subsequently, the separated proteins were transferred onto PVDF membranes (Amersham, Buckinghamshire, UK). These membranes were then subjected to a blocking step employing 5% non-fat dried milk for a duration of 2 h. Following the blocking step, the membranes were incubated with primary antibodies for a period of 12 h. The primary antibodies employed in this investigation included the following: anti-IL-6 antibody (1:1000, Cell Signaling Technology, #12,912), anti-COL2A1 antibody (1:500, Abcam, ab34712), anti-MMP13 antibody (1:1000, Santa Cruz Biotechnology, sc-515,284), anti-p16^INK4a^ antibody (1:1000; Abcam, ab270058), anti-β-actin antibody (1:1000, Cell Signaling Technology, #4970), anti-p-STAT3 antibody (1:1000, Santa Cruz Biotechnology, sc-293,059), anti-STAT3 antibody (1:1000, Santa Cruz Biotechnology, sc-8019), anti-p-JAK1 antibody (1:1500, Abcam, ab138005), and anti-JAK1 antibody (1:1000, Abcam, ab133666). Following the washing step in TBST (composed of 10 mM Tris, pH 8.0, 150 mM NaCl, and 0.1% Tween 20), the membranes were subjected to a 2-h incubation with horseradish peroxidase conjugated anti-rabbit or anti-mouse antibody (1:2000; Abcam, ab205718, ab205719). The process of normalization was conducted utilizing blotting on identical membranes using an antibody against β-actin. The quantification of relative expression was performed employing Quantity One program, specifically version 4.52 (Bio-Rad).

### Establishment of OA Model and Mir-653-5p treatment

The C57BL/6J mice were kept in a controlled environment with a 12-h light/dark cycle. They were provided unlimited access to standard mice food and water. Subsequently, a surgical intervention was conducted in order to produce a model of experimental OA in mice that were 10 weeks old [18]. The procedure included the use of a surgical microscope to accomplish destabilization of the medial meniscus (DMM) in the right knee joints under general anesthesia. In addition, sham procedures were conducted by surgically opening and exposing the right knee structures, followed by the subsequent closure of the articular capsule and skin incision without any disruption to the joint tissue.

For miR-653-5p treatment of experimental OA, 10 µL (200 nmol) volume agomiR-653-5p, antagomiR-653-5p, or their negative controls (GenePharma, China) were administrated into the knee joint employing a 33G needle and a micro-syringe (Hamilton). The mice were treated with their first injection 7 days after DMM. Subsequently, the second and third injections were administered during the second and third weeks after DMM, respectively. The mice were euthanized at the end of an 8-week treatment and then underwent histopathological investigation. The Institutional Animal Care and Use Committee of the University of Southeast authorized all experiments on mice in our investigation procedure.

### Immunohistochemistry (IHC) and histochemical staining

The cartilage specimens were subjected to fixation in a solution containing 4% paraformaldehyde, followed by decalcification employing a solution containing 10% EDTA. The dehydrated specimens underwent embedding in paraffin, followed by the cutting of pieces of 5 mm in thickness. The slides underwent quenching in a solution containing 3% H_2_O_2_ in methanol, followed by three rinses in PBS following the processes of deparaffinization and rehydration. The slides were subsequently exposed to antigen retrieval employing trypsin at 37 °C for 20 min. Following three rinses in PBS, the slides were subjected to a 30-min incubation with a blocking reagent. The slides were subjected to incubation at 4 °C overnight with primary antibodies [MMP3 (1:500, Santa Cruz Biotechnology, sc-21,732); TNF-α (1:1000, Abcam, ab27026)]. The sections next received successive treatment with a biotinylated secondary antibody and a streptavidin-peroxidase conjugate and developed employing a DAB Substrate Kit for peroxide (Vectastain Universal ABC Kit; Vector). The negative control segments were subjected to incubation with an IgG isotype control.

The tissues underwent embedding in paraffin, followed by sectioning and subsequent staining with hematoxylin-eosin and Safranin O/fast green. The decalcified cartilage specimens were subjected to staining with Safranin O and then assessed employing the osteoarthritis research society international (OARSI) grading method with scores ranging from 0 to 6 [19].

### Immunofluorescent analysis

The cultured cells received treatment with a fixative solution consisting of 4% paraformaldehyde. Subsequently, they were permeabilized using a PBS solution comprising 0.25% Triton X-100 for 10 min at room temperature. Nonspecific binding sites were blocked using goat serum. The cells were subjected to overnight incubation with primary antibodies at 4 °C. The main antibodies utilized in the investigation included anti-COL II antibody (1:100, Sigma Aldrich, AB2031), anti-aggrecan antibody (1:1000, Santa Cruz Biotechnology, sc-33,695), anti-MMP13 antibody (1:2000, Abcam, ab39012), anti-ADAMTS5 antibody (1:1000, Abcam, ab246975), anti-IL-6 antibody (1:1000, Cell Signaling Technology, #12,912), anti-p21 antibody (1:100, Abcam, ab109520), and anti-p16^INK4a^ antibody (1:1000; Abcam, ab270058). The cells underwent three rinses with PBS and were then treated with goat anti-rabbit IgG (H&L) combined with either Alexa Fluor 555 (1:100, Abcam, ab150078) or Alexa Fluor 488 (1:500, Abcam, ab150077). Following the washing procedure, the nuclei were then subjected to counterstaining with DAPI (Invitrogen) for 5 min. A confocal microscope (Carl Zeiss, Oberkochen, Germany) was utilized to visualize the fluorescence.

### Statistical analysis

For statistical analysis, the GraphPad Prism 7 program (GraphPad Software, San Diego, CA, USA) was utilized. Normal distribution of the data was verified employing the Shapiro-Wilk test. Comparison of statistical variations between the two experimental groups was detected by two-tailed unpaired Student’s t-test (for parametric data). Statistical analysis was utilized to compare multiple groups with one-way analysis of variance (ANOVA) followed by Tukey’s post hoc, Sidak’s post hoc or Bonferroni’s post hoc test. A *P* value of < 0.05 was considered statistically significant.

## Results

### Identification of miRNAs differentially expressed in degenerative OA cartilage tissues

**Fig. 1:**
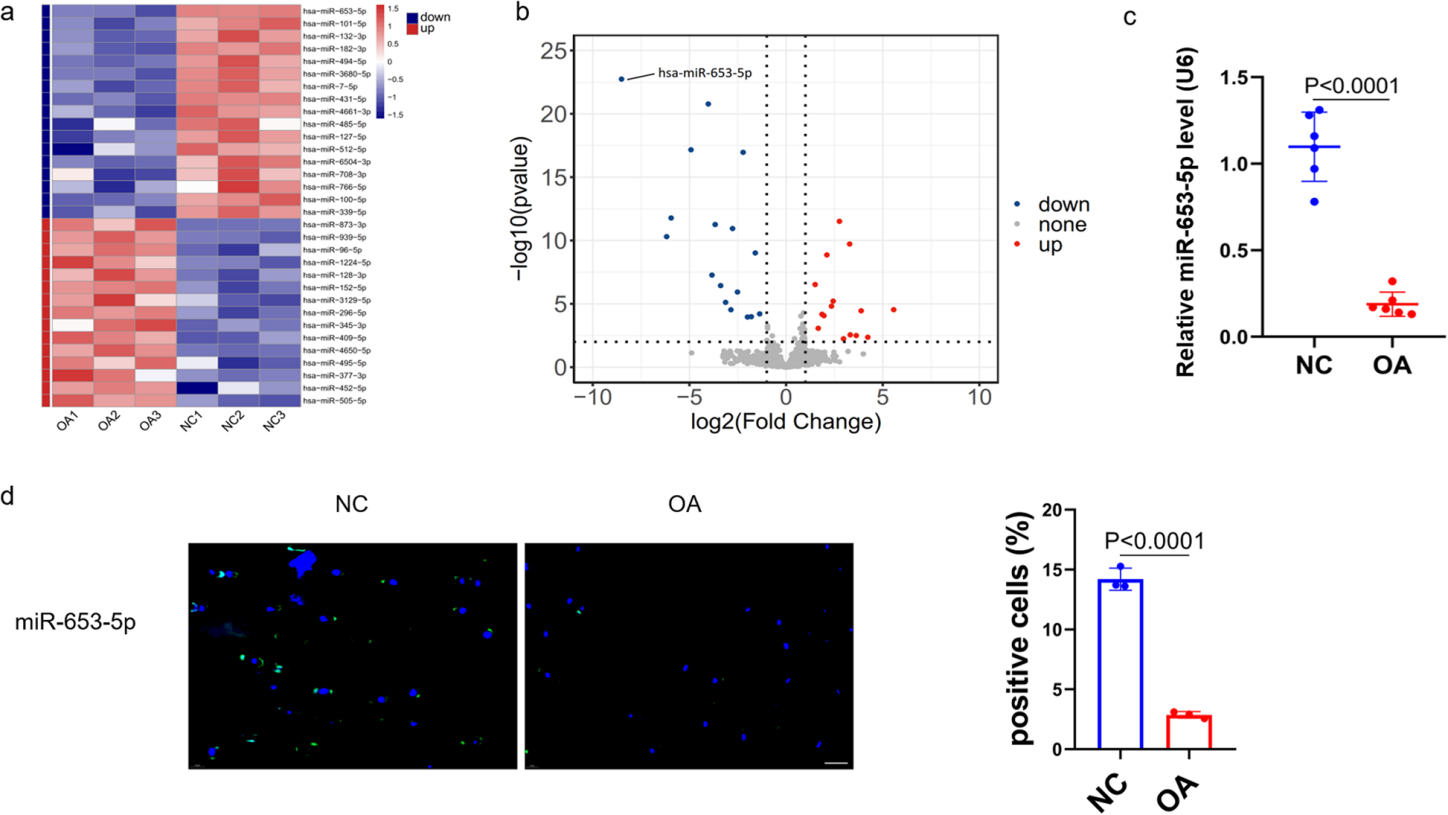
Identification of differentially expressed miRNAs in OA cartilage tissues. **a **Heat map depicting 32 differentially expressed miRNAs between OA and NC. Red represents higher and blue represents lower expression relative to the mean intensity value (white) across all samples (fold change > 2 or < 0.5, Benjamini–Hochberg-corrected p). **b** Volcano plot illustrating the biological and statistical significances of differential miRNA expressions between OA and NC. The negative Log10-adjusted *P* values (y axis) are plotted against the Log2 fold changes in expression (x axis). Red dots indicate the upregulated (right side) and blue dots indicate downregulated (left side) miRNAs. miR-653-5p is indicated. **c **Compared with controls (*n* = 6), miR-653-5p expression level in human chondrocytes was downregulated in OA patients (*n* = 6) using qRT-PCR assay. **d** FISH analysis of chondrocytes from OA patients demonstrated decreased level of miR-653-5p compared to normal controls (*n* = 3). Scale bar = 10 μm. *P* values are from two-tailed unpaired Student’s t-test (*c*, *d*). has, human; FISH, fluorescence in situ hybridisation; miRNA, microRNA; OA, osteoarthritis; NC, normal control

A high-throughput sequencing of miRNA was obtained on three human OA cartilage tissues vs. three human normal cartilage tissues. Using unsupervised clustering analysis, in conjunction with the highly dysregulated miRNAs, facilitated the differentiation between individuals with OA and normal controls. These differentially expressed miRNAs were selected for additional investigation only when they obtained the following measures [20]: (1) having at least 20 miRNA expression copies, (2) mean fold change > 2 or < 0.5, and (3) adjusted *P*-values < 0.01. Depending on these criteria, 32 miRNAs were differentially expressed in patients in comparison to controls, including 15 upregulated and 17 downregulated miRNAs (Fig. 1a, b). Of the several miRNAs that were differentially expressed, miR-653-5p exhibited the most downregulation. The expression of the putative miRNAs was confirmed by employing qRT-PCR test. qRT-PCR results revealed downregulation of miR-653-5p expression level in human chondrocytes from six OA cartilage tissues compared with six controls (Fig. 1c), which was further confirmed by FISH (Fig. 1d). Consequently, we selected miR-653-5p for further investigation.

### Upregulation of miR-653-5p enhances matrix synthesis and cellular proliferation of human chondrocytes and inhibits human chondrocyte senescence

**Fig. 2:**
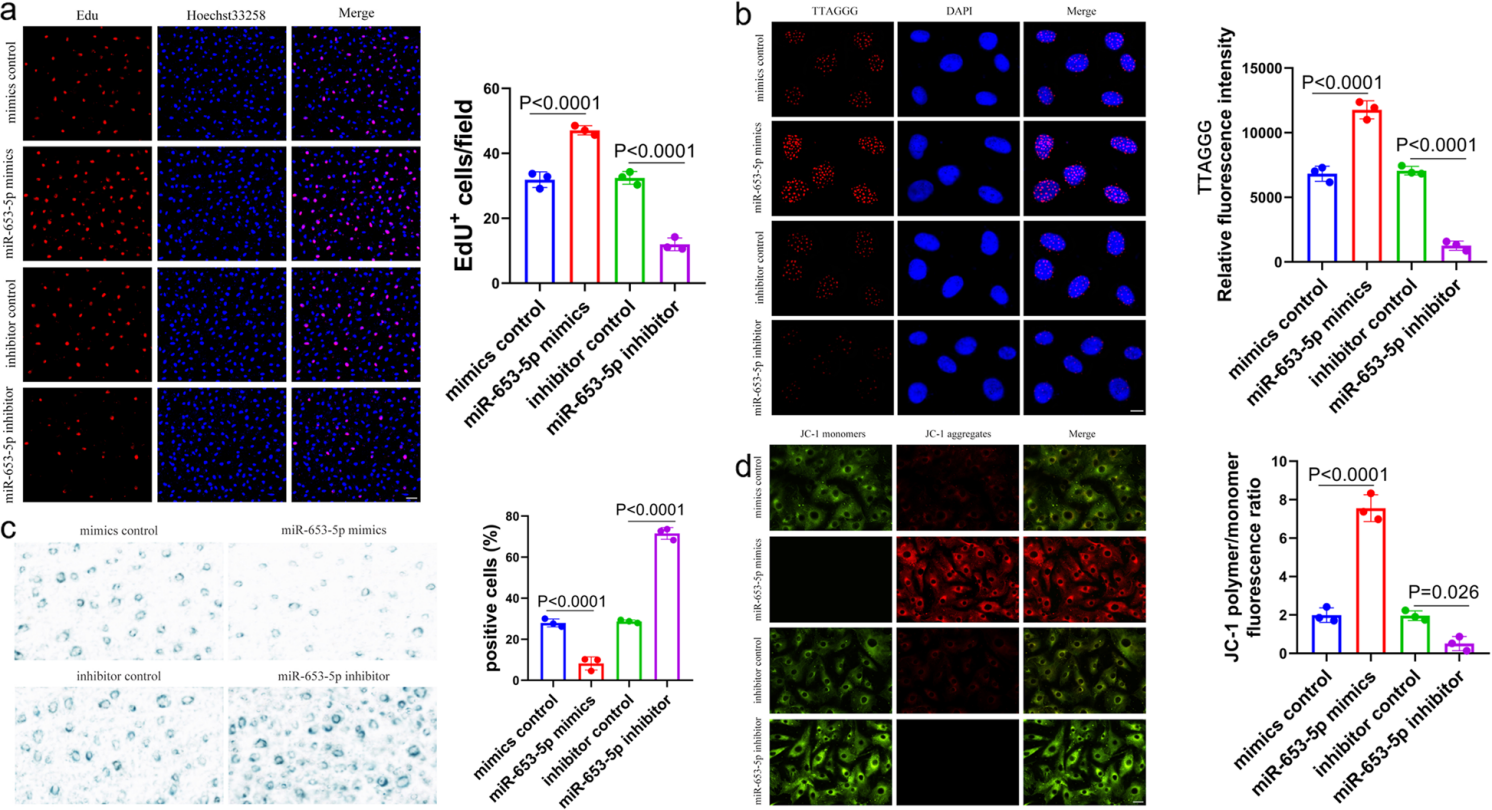
In vitro study of miR-653-5p. **a **Cell proliferation was analyzed in miR-653-5p mimics or inhibitor transfected cultured primary human chondrocytes using EdU assay. *n* = 3 replicates per group. Scale bar = 10 μm. **b **Telomere length analysis by FISH was performed in miR-653-5p mimics or inhibitor transfected cultured primary human chondrocytes. n = 3 replicates per group. Scale bar = 10 μm. **c** SA-β-Gal positivity detection assay was conducted in miR-653-5p mimics or inhibitor transfected cultured primary human chondrocytes. *n* = 3 replicates per group. Scale bar = 20 μm. **d** Analysis of mitochondrial membrane potential was assayed in miR-653-5p mimics or inhibitor transfected cultured primary human chondrocytes. *n* = 3 replicates per group. Scale bar = 10 μm. *P* values are from one-way ANOVA test followed by Tukey’s post hoc test (**a**, **c**) or by Sidak’s post hoc test (**b**) or by Bonferroni’s post hoc test (**d**). miRNA, microRNA; FISH, fluorescence in situ hybridization

**Fig. 3:**
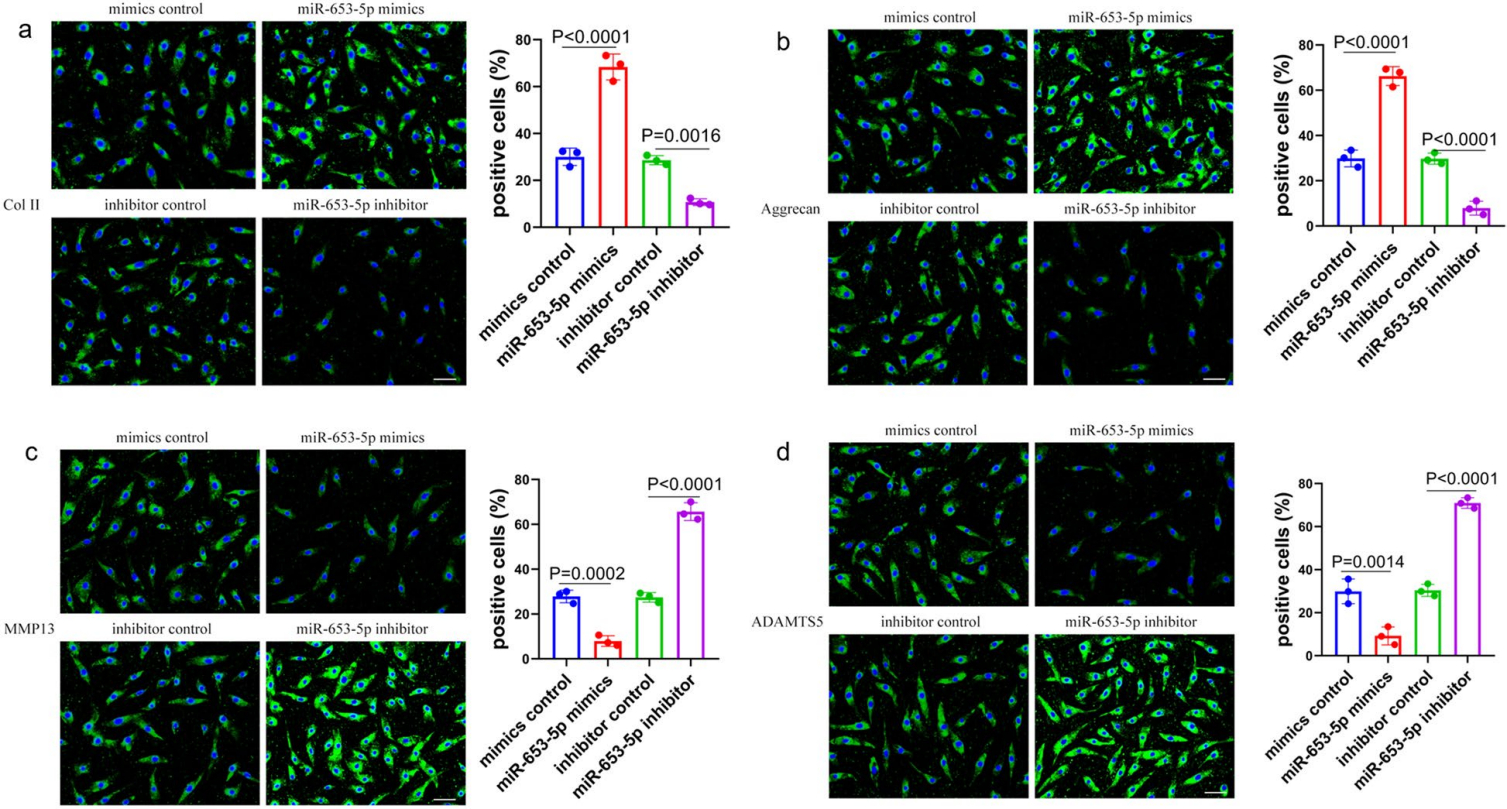
Immunofluorescence study on the effect of miR-653-5p on chondrocytes. **a** The protein expression of Col II was detected by the immunofluorescence in miR-653-5p mimics or inhibitor transfected cultured primary human chondrocytes. *n* = 3 replicates per group. Scale bar = 50 μm. **b** The protein expression of aggrecan were detected by the immunofluorescence in miR-653-5p mimics or inhibitor transfected cultured primary human chondrocytes. *n* = 3 replicates per group. Scale bar = 50 μm. **c** The protein expression of MMP13 was detected by the immunofluorescence in miR-653-5p mimics or inhibitor transfected cultured primary human chondrocytes. *n*  = 3 replicates per group. Scale bar = 50 μm. **d** The protein expression of ADAMTS5 was detected by the immunofluorescence in miR-653-5p mimics or inhibitor transfected cultured primary human chondrocytes. *n* = 3 replicates per group. Scale bar = 50 μm. *P* values are from one-way ANOVA test followed by Bonferroni’s post hoc test (**a**, **b**, **c**, **d**). Col II, collagen type II; MMP13, matrix metallopeptidase 13; ADAMTS5, a disintegrin and metalloproteinase with thrombospondin motifs 5

In order to enhance comprehension of the biological miR-653-5p function in chondrocyte senescence and the pathology of OA, we conducted a transient transfection experiment employing mimic control, miR-653-5p mimic, inhibitor control, or miR-653-5p inhibitor in primary chondrocytes of the human. At 48 h subsequent to the transfection process of the miR-653-5p mimic, cell growth was significantly greater than the miR-653-5p inhibitor, mimic control, and inhibitor control, as proved by an EdU proliferation assay (Fig. 2a). The telomere length experiment, as revealed by FISH staining, demonstrated that the miR-653-5p mimic transfection group exhibited notably enhanced telomere length and integrity, whereas in the miR-653-5p inhibitor group, telomere length and integrity were notably reduced.(Fig. 2b). More notably, human chondrocytes that underwent miR-653-5p inhibitor transfection had decreased mitochondrial membrane potential (Fig. 2d) and raised SA-β-Gal positivity (Fig. 2c). In addition, gain-of-function and loss-of-function investigations were conducted to examine the miR-653-5p expression impact on SASP markers, such as Col II, aggrecan, MMP13, and ADAMTS5, in human chondrocytes by employing the technique of immunofluorescence staining. As illustrated in Fig. 3a and b, miR-653-5p overexpression in chondrocytes powerfully elevated levels of Col II and aggrecan, whereas suppression of miR-653-5p diminished Col II and aggrecan levels. In contrast to the expression of cartilage matrix synthesis biomarkers, the MMP13 and ADAMTS5 expression was reduced in chondrocytes with miR-653-5p overexpression in contrast to the suppression expression of miR-653-5p (Fig. 3c, d). Collectively, the data indicated that the upregulation of miR-653-5p could promote chondrocyte matrix synthesis and proliferation and inhibit chondrocyte senescence.

### Verification of IL-6 as a target gene for miR-653-5p

**Fig. 4:**
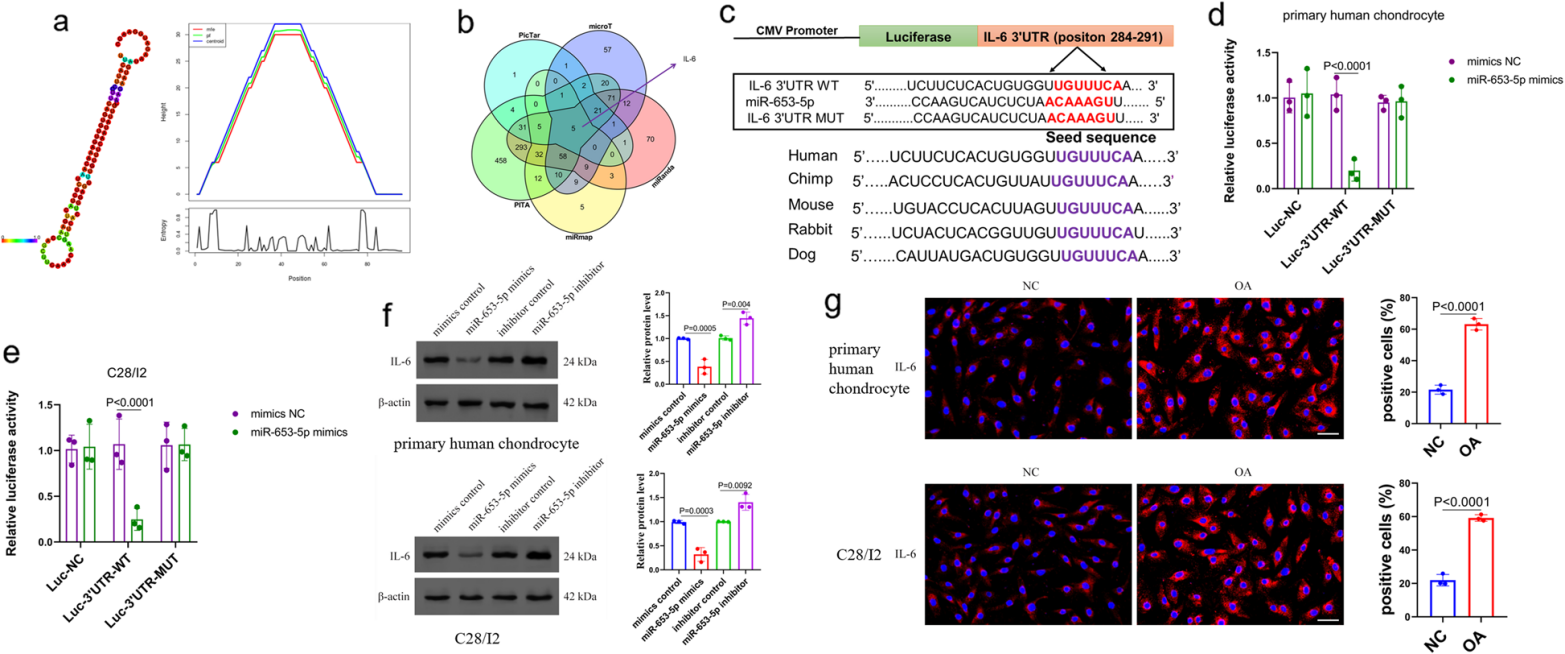
Identification of IL-6 as a target of miR-653-5p. **a **The secondary structure and the positional entropy for each position of miR-653-5p. **b** Venn diagram displaying miR-653-5p computationally predicted to target IL-6 by different algorithms. **c** Schematic representation of IL-6 3′ UTR demonstrating putative miRNA target site, luciferase activities of wild type (WT-UTR) and mutant (MUT-UTR) constructs. **d**, **e** The wild or mutant type IL-6 3′ UTR reporter plasmid was co-transfected with mimic-NC or miR-653-5p mimic into human primary chondrocytes and C28/I2 cells. 48 h after transfection, luciferase activity was measured. *n* = 3 replicates per group. **f** Western blot assays showed that overexpression of miR-653-5p could decrease IL-6 protein level, while inhibition of miR-653-5p could increase IL-6 protein level in both primary human chondrocytes and C28/I2 cells. *n* = 3 replicates per group. **g** Compared with normal controls, IL-6 protein expression was increased in human primary OA chondrocytes and C28/I2 OA cells using the immunofluorescence analysis. *n* = 3 replicates per group. Scale bar = 50 μm. *P* values are from one-way ANOVA test by Tukey’s post hoc test (**d**, **e**, **f**) and two-tailed unpaired Student’s t-test (**g**). miRNA, microRNA; IL-6, interleukin 6; OA, osteoarthritis; NC, normal control

We investigated the secondary structure and positional entropy of miR-653-5 at each site (Fig. 4a). Identifying potential targets of miR-653-5p through miRanda (http://www.microrna.org), DIANA-microT (https://bio.tools/DIANA-microT), PicTar (http://pictar.mdc-berlin.de), miRmap (https://mirmap.ezlab.org), and PITA (http://genie.weizmann.ac.il/pubs/mir07/mir07), IL-6 was identified as the target of miR-653-5p (Fig. 4b). In order to provide more evidence on the functional interaction between miR-653-5p and IL-6, luciferase reporter examinations were conducted employing an IL-6 vector. This vector included either the putative binding sites for miR-653-5p (referred to as wild type, WT) or mutant binding sites (MUT) located in the 3′UTR (Fig. 4c). As shown in Fig. 4c, the high degree of conservation in the seed sequence of miR-635-5p binding to IL-6 across different species made the miR-635-5p intervention treatment stable and uniform in different experimental models. The relative luciferase reporter activity of human primary chondrocytes and C28/I2 cells, when co-transfected with IL-6 (WT) and mimic of miR-653-5p, exhibited a significantly lower level compared to the relative luciferase reporter activity seen in human primary chondrocytes and C28/I2 cells underwent IL-6 (MUT) and miR-653-5p mimic transfection (Fig. 4d, e). This effect was further confirmed by western blot and the immunofluorescence analysis. Figure 4f and S1 showed that miR-653-5p overexpression reduced IL-6 protein expression in both C28/I2 cells and human primary chondrocytes, whereas miR-653-5p suppression increased the level of IL-6 protein. Moreover, IL-6 protein expression was shown to be elevated in both human primary OA chondrocytes and C28/I2 OA cells as compared to normal control cells (Fig. 4g). Collectively, the findings indicated that miR-653-5p recognized the 3′-UTR of IL-6 transcripts directly and modulated its expression after the transcription.

### MiR-653-5p affects the pathological process of OA by regulating chondrocyte senescence through IL-6/JAK/STAT3 signaling pathway

**Fig. 5:**
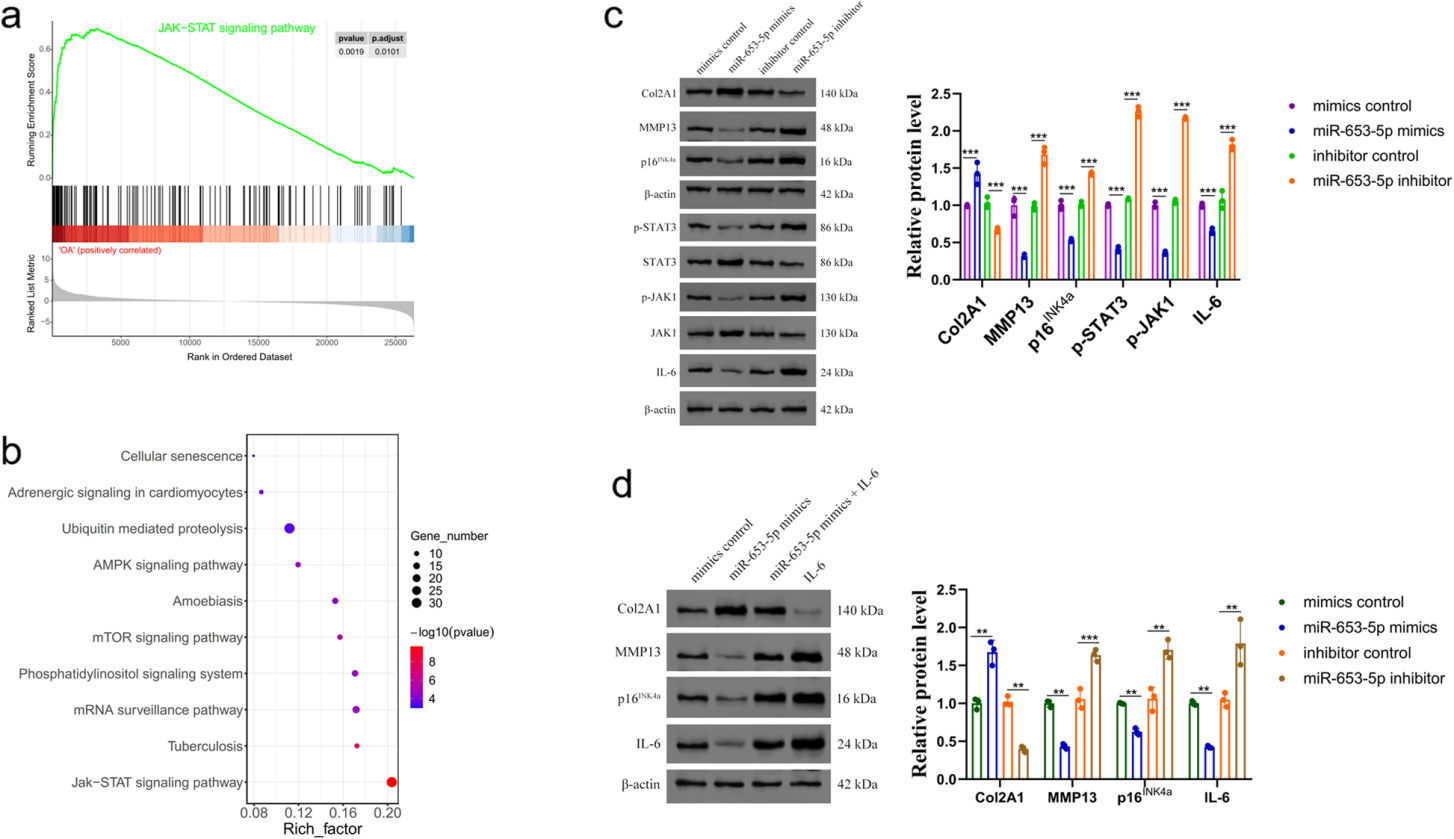
The modulation of miR-653-5p on IL-6/JAK/STAT3 signaling pathway. **a **GSEA analysis demonstrating JAK/STAT signaling pathway enriched in OA. **b **KEGG analysis demonstrating JAK/STAT pathway enriched in OA. **c **Cultured primary human OA chondrocytes were transfected with miR-653-5p mimics, miR-653-5p inhibitor and their negative controls for 72 h and then the levels of IL-6, JAK1, p-JAK1, STAT3, p-STAT3, p16 INK4a , MMP13 and Col2A1 were measured by western blotting. Quantitative analysis was shown on the right, n  = 3 independent biological replicates per group. * * * *p* < 0.001 by one-way ANOVA test followed by Tukey’s post hoc test. **d** The rescue experiment was established in cultured primary human OA chondrocytes to validate the relationship between miR-653-5p and IL-6. Inhibition of MMP13, p16 INK4a and IL-6 expression levels by miR-653-5p mimics was rescued by restoration of IL-6 expression. In comparison, inhibition of Col2A1 expression by IL-6 overexpression was rescued by miR-653-5p mimics. Quantitative analysis was shown on the right, *n* = 3 independent biological replicates per group. * * *p* < 0.01, * * * *p* < 0.001 by one-way ANOVA test followed by Tukey’s post hoc test. miRNA, microRNA; IL-6, interleukin 6; Col, collagen; MMP13, matrix metallopeptidase 13; JAK, janus kinase; OA, osteoarthritis; NC, normal control

Figure 5a and b showed that in GSEA and KEGG analysis, the signaling pathway of JAK/STAT3 was significantly upregulated and enriched. IL-6 is a well-recognized activator of cytokines within the pathway of JAK/STAT, and raised concentrations of IL-6 have been identified in chronic inflammatory disorders, including rheumatoid arthritis and OA [21]. To discover whether miR-653-5p exerted its roles through the pathway of JAK/STAT3, which contributed to chondrocyte senescence and progression of OA, we studied the major components transcription (IL-6, JAK1, p-JAK1, STAT3, p-STAT3) of the IL-6/JAK/STAT3 pathway, as well as the senescence phenotypes and cartilage matrix biomarkers (p16^INK4a^, MMP13, Col2A1) expression. Primary human OA chondrocytes that had been cultured were subjected to transfection with miR-653-5p mimics, miR-653-5p inhibitor, or their corresponding negative control, respectively. Col2A1, STAT3, and JAK1 expression levels were significantly increased, and MMP13, p16^INK4a^, p-STAT3, p-JAK1, and IL-6 expression were downregulated in human OA chondrocytes that were stably overexpressed miR-653-5p (Fig. 5c, S2). In contrast, MMP13, p^16INK4a^, p-STAT3, p-JAK1, and IL-6 expression levels were upregulated in human OA chondrocytes transfected with miR-653-5p inhibitor (Fig. 5c, S2). Additional rescue experiments were conducted to prove the link between IL-6/JAK/STAT3 and miR-653-5p. MMP13, p16^INK4a^, and IL-6 expression levels were suppressed by the introduction of miR-653-5p mimics. However, this inhibition was rescued when IL-6 expression was restored. In contrast, the rescue of Col2A1 expression inhibition caused by IL-6 overexpression was observed with the introduction of miR-653-5p mimics (Fig. 5d, S2). These outcomes indicated that miR-653-5p could suppress OA progression through IL-6/JAK/STAT3 signaling pathway modulation.

### Therapeutic effects of mir-653-5p as a potential target in a mouse DMM model

**Fig. 6:**
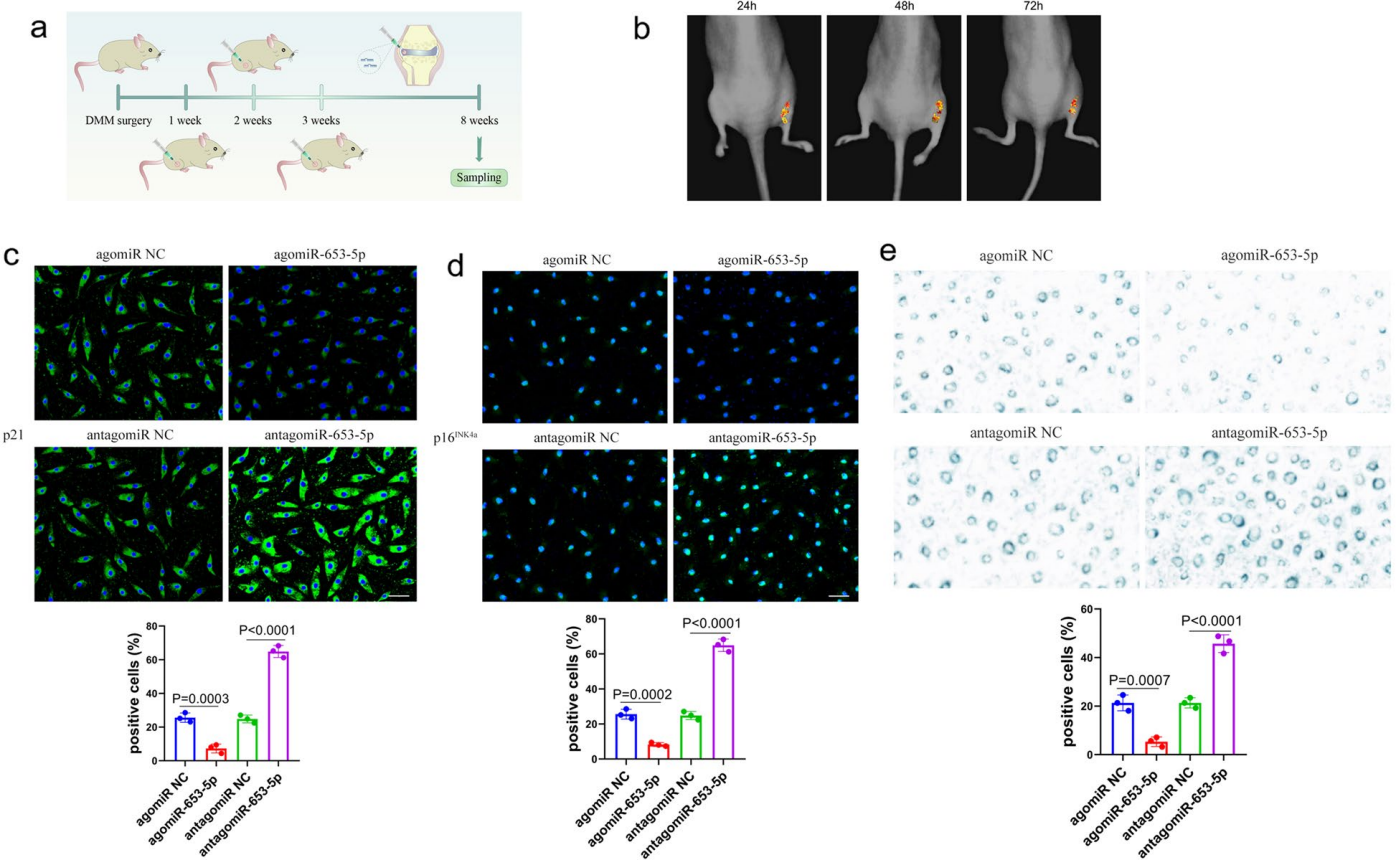
Effect of miR-653-5p on chondrocyte senescence in DMM animal models. **a **An overview of the experimental set-up with injections of agomiR-653-5p, antagomiR-653-5p or their negative controls at 1, 2, and 3 weeks after DMM surgery. **b **In vivo time-dependent fluorescence images in mice at 24, 48, and 72 h after the administration of Cy3-miR-653-5p. **c**, **d** Immunofluorescence for p21 and p16 INK4a from the chondrocytes in DMM model treated by agomiR-653-5p, antagomiR-653-5p or their negative controls at 8 weeks. Scale bar = 50 μm. *n* = 3 mice per group. **e** SA-β-Gal positivity was analyzed from the chondrocytes in DMM model treated by agomiR-653-5p, antagomiR-653-5p or their negative controls at 8 weeks. *n* = 3 mice per group. *P* values are from one-way ANOVA test followed by Bonferroni’s post hoc test (**c**, **d**, **e**). miRNA, microRNA; DMM, destabilization of the medial meniscus; NC, normal control

**Fig. 7:**
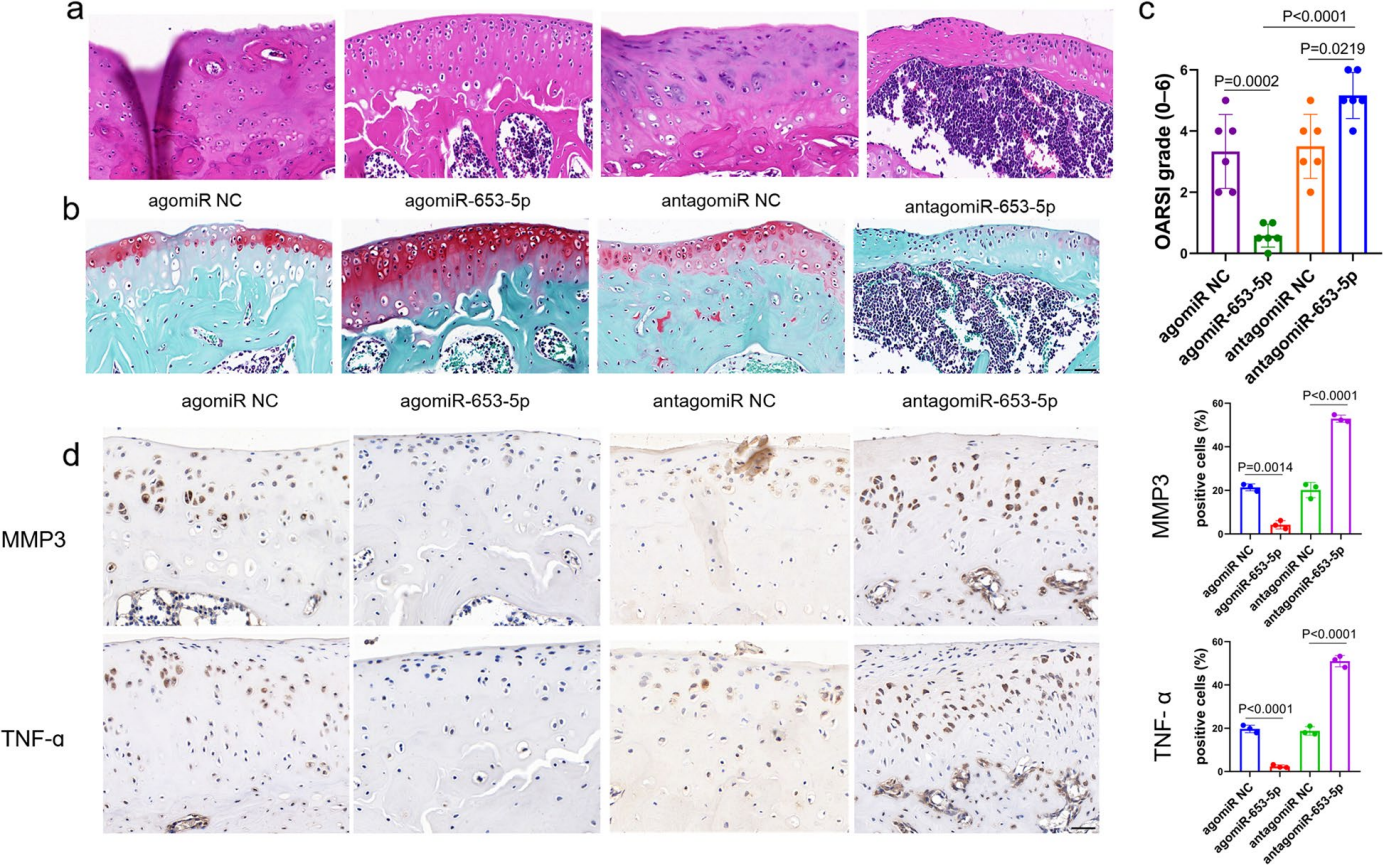
Effect of miR-653-5p on OA progression in DMM animal models. **a **At 8 weeks following DMM surgery, the articular cartilage degeneration was evaluated by H&E staining in different groups treated by agomiR-653-5p, antagomiR-653-5p or their negative controls. *n* = 6 mice per group. Scale bar = 50 μm. **b**, **c** Safranin O-fast green stained sections showed that OA phenotype was significantly alleviated in the DMM-induced OA mice model treated by agomiR-653-5p, as evaluated by OARSI. *n* = 6 mice per group. **d** Immunostaining for MMP3 and TNF-α in the DMM model treated by agomiR-653-5p, antagomiR-653-5p or their negative controls at 8 weeks. *n* = 3 mice per group. Scale bar = 50 μm. *P* values are from one-way ANOVA test followed by Tukey’s post hoc test (**c**) and by Bonferroni’s post hoc test (**d**). miRNA, microRNA; DMM, destabilization of the medial meniscus; MMP3, matrix metallopeptidase 3; TNF-α, tumor necrosis factor-α; OA, osteoarthritis; NC, normal control; OARSI, osteoarthritis research society international

To discover the therapeutic impact of miR-653-5p in vivo, a mouse model of DMM was created, followed by local intra-articular (IA) administration of agomiR-653-5p, antagomiR-653-5p, or their negative controls at 1, 2, and 3 weeks following the surgical procedure (Fig. 6a). Moreover, in vivo Cy3-labeled miR-653-5p fluorescence analysis demonstrated that miR-653-5p could persist in the joint cavity for at least 72 h (Fig. 6b). Immunofluorescence analysis revealed a significant reduction in p21 and p16^INK4a^ expression in chondrocytes from the cartilage of agomiR-653-5p treated DMM model compared to those treated with antagomiR-653-5p or their respective negative controls, at the 8-week time point (Fig. 6c, d). In addition, a decrease in SA-β-Gal positivity was observed in chondrocytes from the cartilage of the DMM model treated with agomiR-653-5p compared to those treated with antagomiR-653-5p or their respective negative controls at 8 weeks (Fig. 6e). At 8 weeks, the articular cartilage degeneration was assessed by staining with H&E and Safranin O-fast green in different groups. Results exhibited that in the DMM-induced OA mice model treated by agomiR-653-5p, the OA phenotype was significantly diminished, as evaluated by the scoring system of OARSI on the medial aspect of the joint (Fig. 7a, b, c). Notably, IA injection of agomiR-653-5p remarkably decreased SASP factors (MMP3 and TNF-α) in DMM-operated mice, as opposed to control injections (Fig. 7d). These findings implied that miR-653-5p is a potential therapeutic target for OA.

## Discussion

**Fig. 8:**
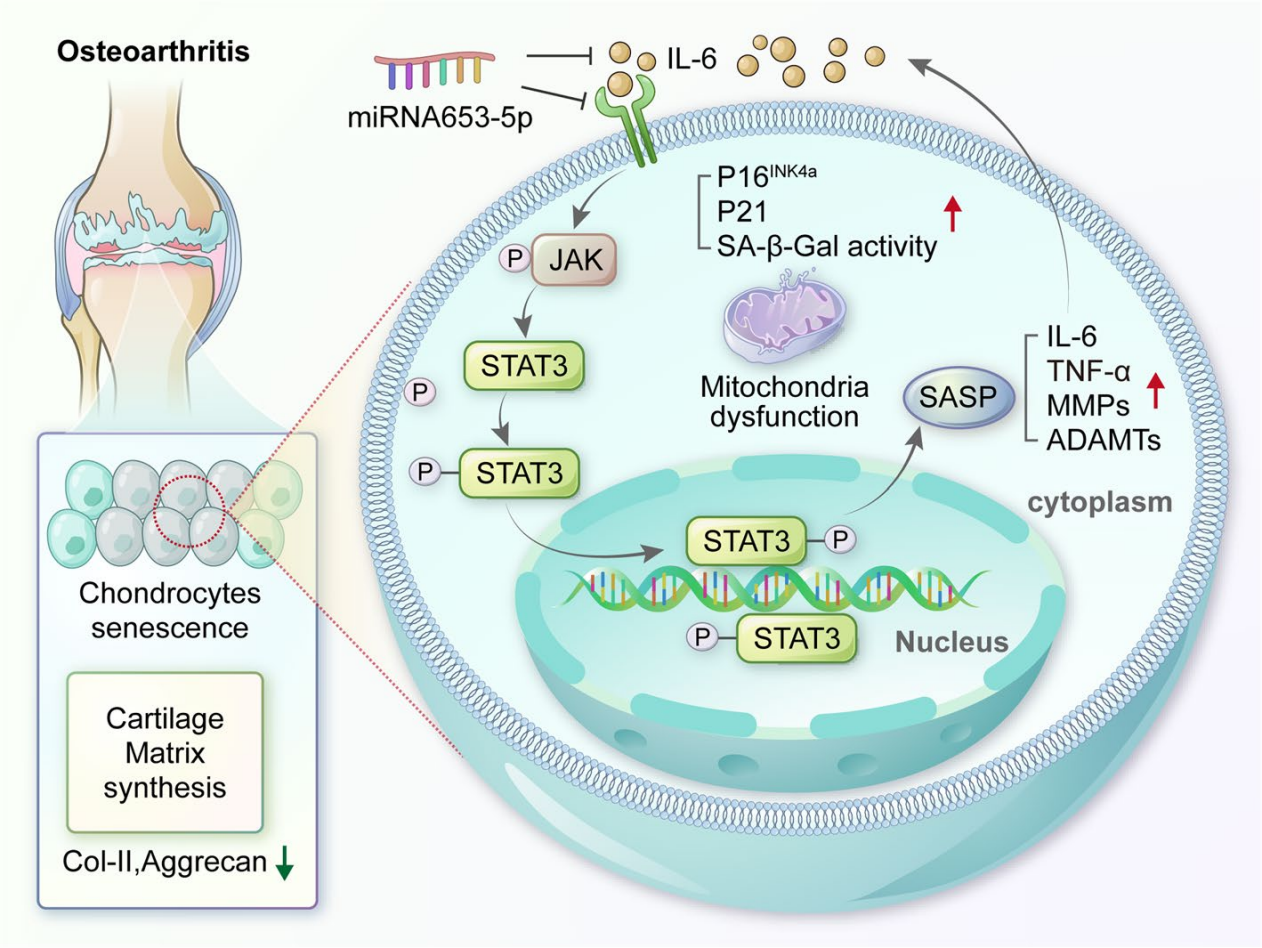
Schematic diagram of the effect of miR-653-5p on chondrocyte senescence during the pathogenesis of osteoarthritis

In the present study, we demonstrated for the first time that miR-653-5p was markedly downregulated in cartilage tissues and chondrocytes from OA patients and were the first to identify IL-6 as a target of miR-653-5p. We showed that IL-6 expression was substantially downregulated by miR-653-5p, which in turn inhibited chondrocyte senescence and alleviated cartilage degeneration through IL-6/JAK/STAT3 signaling (Fig. 8). Our findings proved in a DMM animal model that IA injection of miR-653-5p is a potential therapeutic approach for preventing and treating OA.

For decades, researchers are trying to find targets that can reverse or delay the progression of OA by revealing the pathogenesis of OA, to treat OA effectively. Recently, cell senescence, an “ancient” topic, has re-entered the region of interest of scholars. The evidence implicating cellular senescence in cartilage as a primary driver of OA pathogenesis and progression is compelling, however, the exact mechanism linking senescence to OA pathology remains unclear [3]. In a recent study by our research group, we found that Sirt6 attenuated chondrocyte senescence by inhibiting IL-15/JAK3/STAT5 signaling and disruption in this signaling contributed to the OA progression [22]. This finding confirms that regulating chondrocyte senescence is dramatically meaningful and will have the opportunity to change the process of OA.

A growing body of evidence demonstrates that miRNAs are dysregulated in cartilage during OA and modulating specific miRNAs in cartilage may be a novel therapy in the treatment of OA [23]. For instance, miR-140 expression is reduced in OA cartilage compared to healthy cartilage. IA administration of miRNA-140 significantly alleviates OA progression by maintaining cartilage homeostasis [24]. Our previous study also showed that miR-218-5p was a novel inducer of cartilage destruction via modulation of PI3K/Akt/mTOR signaling [25]. Notably, our data showed that miR-653-5p expression was significantly reduced in OA cartilage and chondrocytes compared to healthy cohorts. In fact, senescent joint cells manifest shared characteristics, including telomere erosion, increased expression of the cyclin-dependent kinase inhibitors p21 and p16^INK4a^, enhanced generation of reactive oxygen species via mitochondrial dysfunction, increased SA-β-gal production, and increased harmful secretion of pro-inflammatory SASPs [26]. In this study, we confirmed that overexpression of miR-653-5p promoted the proliferation of human chondrocytes and the expression of cartilage synthetic matrix (Col II and aggrecan) in vitro, while suppression of miR-653-5p expression reduced the integrity of human chondrocyte telomerase, disrupted mitochondrial function, and increased the expression of cellular senescence phenotypes (SA-β-Gal positivity, MMP13, and ADAMTS5). These findings suggest a strong correlation between the expression of miR-653-5p and chondrocyte senescence.

IL-6 is a pleiotropic pro-inflammatory cytokine involved in many physiological and pathological processes and signal transducer and activator of transcription 3 (STAT3) is the main signaling factor downstream of IL-6 [27]. Accumulating evidence suggests that the IL-6/STAT3 signaling pathway is now considered a critical target for alleviating cartilage damage during OA [28]. Recently, Latourte et al. used a neutralizing antibody of the IL-6 receptor to systemically inhibit the expression of IL-6 and observed an alleviating effect in a DMM-induced OA model [29]. However, our previous studies have indicated that the IL-6/STAT3 signaling pathway also plays essential roles in the development and maintenance of articular cartilage homeostasis [30]. Considering the complexity of gene regulation, miRNAs are emerging as valuable therapeutic candidates, particularly in the context of diseases characterized by multifactorial origins rather than a singular genetic link [31]. In this study, we predicted putative target genes of miR-653-5p using five widely used databases. Moreover, we reconfirmed IL-6 as a target gene regulated by miR-653-5p through the luciferase reporter assay, gain/loss of function of miR-653-5p studies by western blot and immunofluorescence analysis using two cell types (primary human chondrocytes and C28/I2 cells). In addition, we demonstrated that the main components of the IL-6/JAK/STAT3 signaling pathway were regulated by miR-653-5p. We found that miR-653-5p overexpression would inhibit the protein expression of IL-6 and the phosphorylation of JAK1 and STAT3 in chondrocytes, thereby inhibiting the protein expression of MMP13 and p16^INK4a^ while promoting the expression of Col2A1. Furthermore, this process of regulation could be rescued by restoration of IL-6 expression. Together, these findings indicate that miR-653-5p’s capacity to regulate the IL-6/JAK/STAT3 signaling pathway might contribute to the observed chondrocyte senescence and cartilage degradation in OA.

Concerning its therapeutic potential, we noted that treatment of the miR-653-5p agonist markedly protected cartilage from damage in a DMM OA model as assessed by the OARSI scores. Interestingly, the IA injection of agomiR-653-5p treatment decreased the expression of MMP3 and TNF-α, two major components of OA catabolic and inflammatory factors, and suppressed the chondrocyte senescence phenotypes including p21, p16^INK4a^, and the SA-β-Gal activity. Although various anti-cellular senescence agents, namely, senolytics and senomorphics, have been developed, there have not shown satisfactory outcomes in OA treatment till now [32]. Targeting of dysregulated miRNA holds promise as a well-tolerated therapeutic intervention, given that differentially expressed miRNAs are typically disease-specific and do not assume critical roles under normal physiological conditions in adult tissues or in quiescent cells [33]. Moreover, the enhancement of target specificity and efficacy, along with the reduction of side effects, can be achieved through the intralesional administration of miRNA drugs directly into the pathogenic site [34]. Based on our findings, the development of miR-653-5p-based therapeutics for the treatment of OA may have prospects in the future. Moreover, this study has some limitations. Given that a single miRNA can target many mRNAs and that each mRNA can be the target for several miRNAs, it is clear that really understanding IL-6/JAK/STAT3 signaling pathway between chondrocyte senescence and OA will require further research. It is well known that the senescent microenvironment in the OA joint includes not only senescent chondrocytes but also synovial fibroblasts and macrophages. In the future, further research is needed to elaborate on the connection and interaction between different cell types.

## Conclusions

In conclusion, our investigations revealed that miR-653-5p was significantly decreased in cartilage tissues from individuals with OA, causing the upregulation of the chondrocyte senescence phenotypes in the articular cartilage. AgomiR-653-5p might be a probable treatment approach for OA. The comprehension of the IL-6/JAK/STAT3 signaling pathway has the potential to provide logical approaches for medical therapy in individuals with OA.

### Supplementary Information

**Supplementary Material 1.**

## Data Availability

No datasets were generated or analysed during the current study.

## Abbreviations

OA: Osteoarthritis
miRNA: microRNAs
FISH: Fluorescence in situ hybridization
SASP: Senescence-associated secretory phenotype
qRT-PCR: Quantitative real-time PCR
EdU: 5-ethynyl -2′-deoxyuridine
SA-β-Gal: Senescence-associated-galactosidase
IL-6: Interleukin 6
Col II: Collagen type II
MMP: Matrix metallopeptidase
ADAMTS5: A disintegrin and metalloproteinase with thrombospondin motifs 5
TNF-α: Tumor necrosis factor-α
STAT: Signal transducer and activator of transcription
JAK: Janus kinase
DMM: Destabilization of the medial meniscus
OARSI: Osteoarthritis research society international
DAPI: 4,6-diamidino-2-phenylindole
