# Supplementary material for: MiR-653-5p drives osteoarthritis pathogenesis by modulating chondrocyte senescence
Source: Arthritis Res Ther. 2024 May 29;26:111. doi: 10.1186/s13075-024-03334-5 (PMC11134905; doi:10.1186/s13075-024-03334-5)
Supplement: Supplementary file 1 — Supplementary Material 1. [file 13075_2024_3334_MOESM1_ESM.docx]

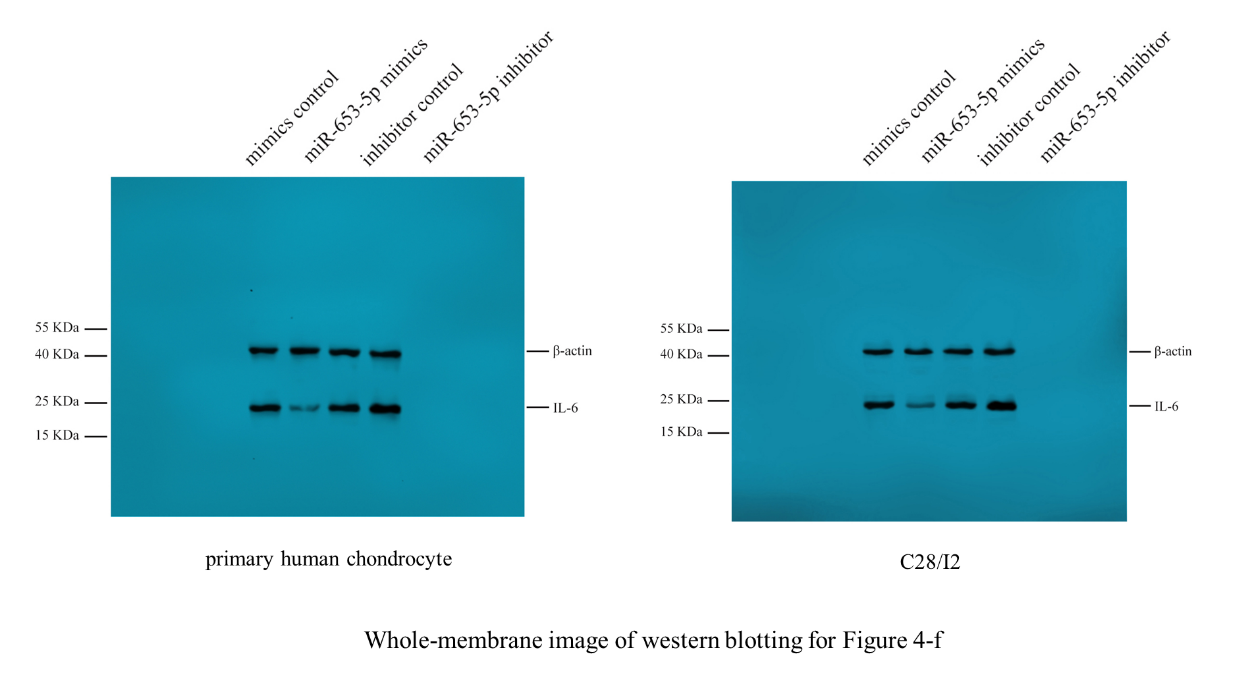


**FIGURE S1 Whole-membrane image of western blotting for Figure 4-f**


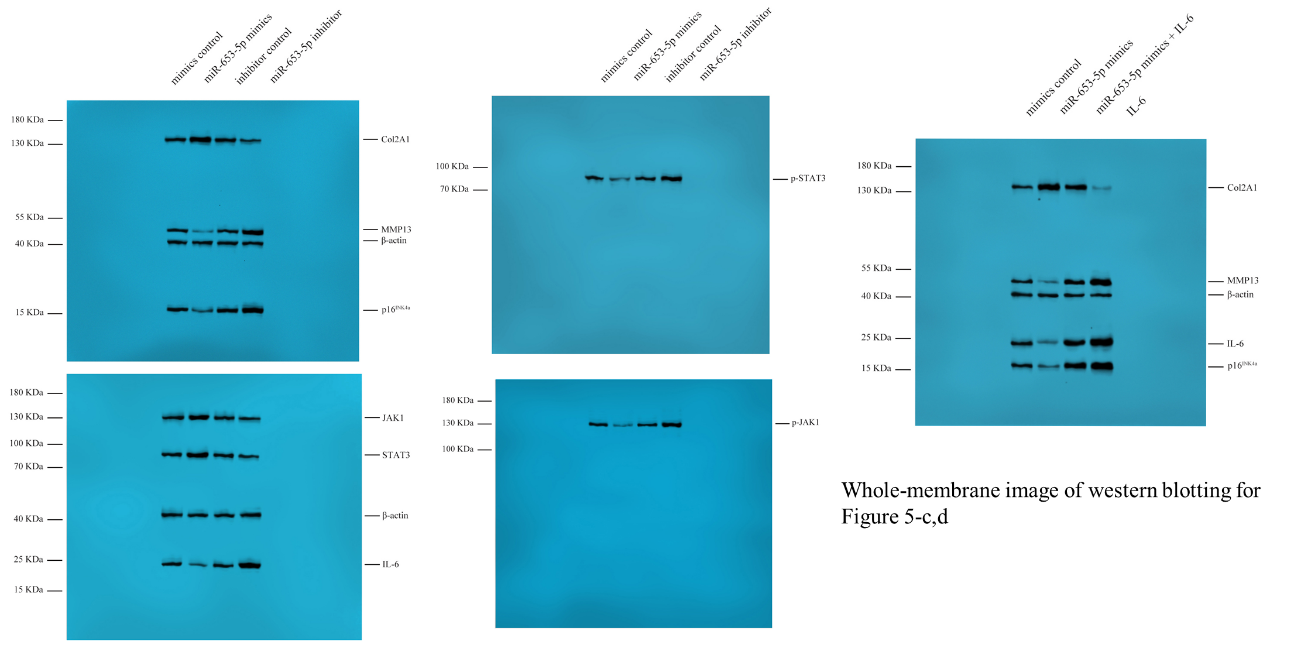


**FIGURE S2 Whole-membrane image of western blotting for Figure 5-c,d**


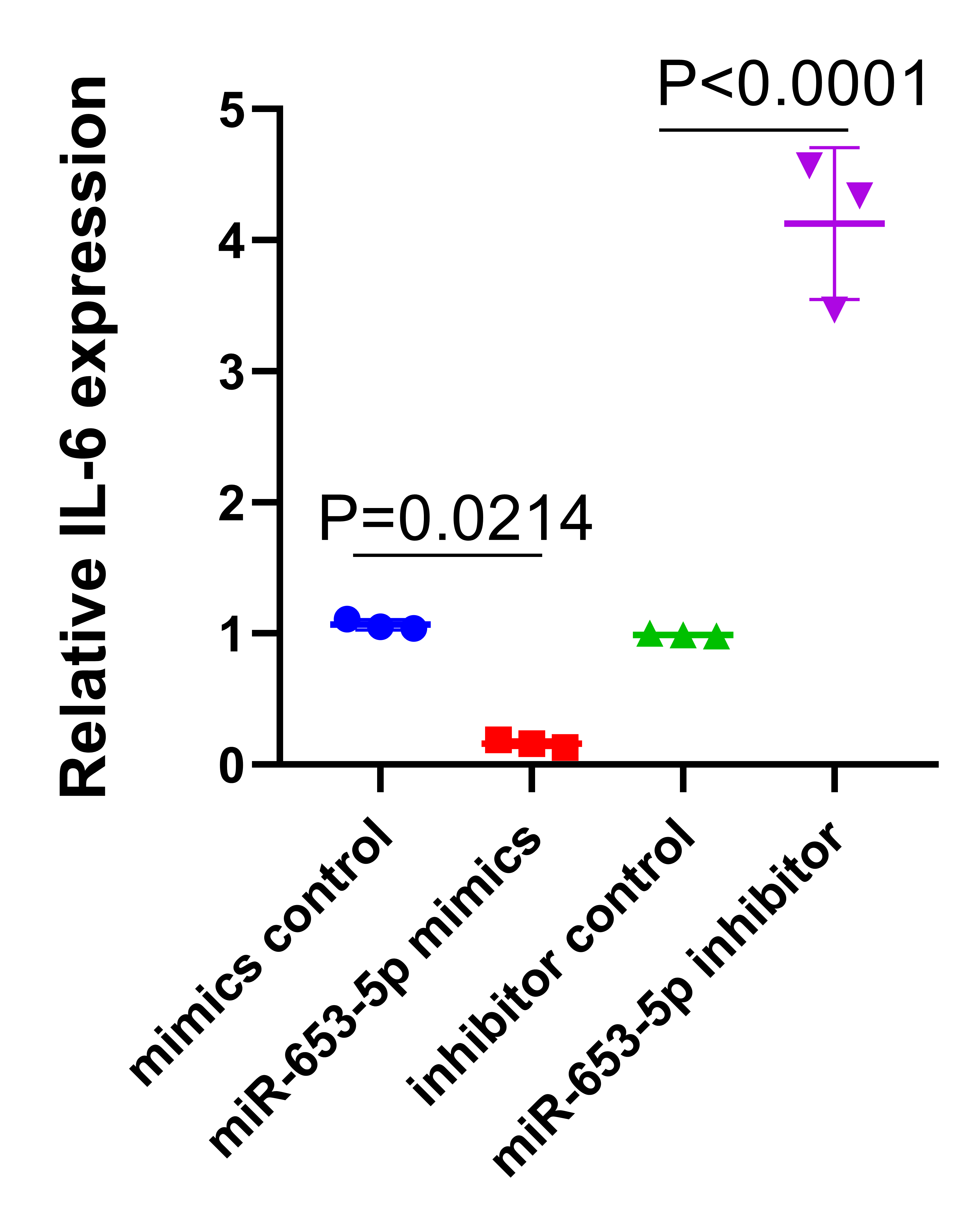


**FIGURE S3** **qRT-PCR experiments to verify the effect of miR-653-5p on IL-6 at the transcription level**

qRT-PCR assays showed that overexpression of miR-653-5p could decrease IL-6 mRNA level, while inhibition of miR-653-5p could increase IL-6 mRNA level in primary human chondrocytes. P values are from one-way ANOVA test followed by Tukey’s post hoc. n = 3 mice per group.
